# Biosafety Initiatives in BMENA Region: Identification of Gaps and Advances

**DOI:** 10.3389/fpubh.2016.00044

**Published:** 2016-03-24

**Authors:** Erum Khan, Nayla Ahmed, Khalid R. Temsamani, Atef El-Gendy, Murray Cohen, Ariba Hasan, Hilliard Gastfriend, Jennifer Cole

**Affiliations:** ^1^Aga Khan University Karachi, Karachi, Pakistan; ^2^Faculté des Sciences de Tétouan Mhannech II, University Abdelmalek Essaadi, Tétouan, Morocco; ^3^Bacteriology Section, Naval Medical Research Unit 3 (NAMRU-3), Cairo, Egypt; ^4^Frontline Foundation, Atlanta, GA, USA

**Keywords:** biosafety, biosecurity code of conduct, survey research, BMENA region, biorisk management

## Abstract

**Introduction:**

The objectives of this study were to identify and assess the impact of capacity-building biosafety initiatives and programs that have taken place in the broader Middle East and North Africa (BMENA) region between 2001 and 2013, to highlight gaps that require further development, and to suggest sustainable ways to build cooperative regional biosafety opportunities.

**Methods:**

A cross-sectional study was conducted with two aspects (1) thorough desktop review of literature for all biosafety/biosecurity-related activities in the study countries, such as seminars, conferences, workshops, policy documents, technology transfer, sustained scientific endeavors between countries, etc. and (2) an online survey of scientists in countries in the region to get first-hand information about biosafety and biosecurity initiatives and gaps in their country.

**Results:**

A total of 1832 initiatives of biosafety/biosecurity were recorded from 97 web links; 70.68% (*n* = 1295) initiatives were focused on raising general awareness among the scientific community about biosafety/biosecurity/biocontainment. The most frequent areas of interest were biorisk management in biomedical and biotechnology laboratories 13% (*n* = 239), followed by living modified organisms (LMOs) 9.17% (*n* = 168). Hands-on training accounted for 2.67% (*n* = 49) of initiatives. Online survey results confirmed desktop review findings; however, the response rate was 11%.

## Introduction

Recent advances in biotechnology have provided a quantum leap in the application of biological sciences in all fields, including health, agriculture, environment, and energy development. Biotechnology tools and protocols are globally available and are increasingly being used and seen as potential investments. However, with rapid advancement, widespread use, and investment, comes the possibility of exploitation of research and risk of misuse of the research outcomes for nefarious purposes. The most concerning issue remains the lack of expertise and awareness of risk management systems for the mitigation of such risks in the biological sciences, despite extensive and well-developed relevant engineering and scientific methods for containment, detection, diagnosis, treatment, and recovery as well as human behavior and performance science in other fields, such as aviation, nuclear power, petrochemicals, etc. This concern has been raised globally and has intensified since the events of 9/11 and the anthrax-in-the-mail terrorism of 2001. This has led to international focus on means for combating bioterrorism, especially in countries with the backdrop of disturbed geopolitical situations (1–5).

This study was undertaken to identify and assess the impact of capacity-building biosafety initiatives and programs that have taken place in broader Middle East and North Africa (BMENA) region between 2001 and 2013, and to highlight gaps that require further development. The project had two components: (a) a review of biosafety and biosecurity initiatives in the region and (b) assessment of the impact of these initiatives on scientists at local/regional levels. This study was part of a larger project titled “*Scientific Engagement Defining Gaps and Creating Opportunities for Cooperative Research and Global Security in the Broader Middle East and North Africa Region*” to assess the overall impact of global biosecurity capacity-building initiatives undertaken in recent years in the BMENA region and to then apply the knowledge gained from this assessment to suggest sustainable ways to build cooperative regional biosafety opportunities.

## Materials and Methods

The project co-PIs, based in Pakistan and the U.S., worked closely with two Regional Coordinators in North Africa (Morocco and Egypt) to recruit a project coordinator in each of the 24 countries participating in the project. A cross-sectional descriptive study was designed consisting of two steps: (1) a desktop review/literature search for all biosafety- and biosecurity-related initiatives that were reported from the BMENA region between 2001 and 2013 using commercial search engines and (2) building a survey instrument in the commercially available SurveyMonkey (SM) platform and administering it independently to scientists in the study countries.

A database was created according to the type of initiatives each study country in the region had, the scope of the activity, institute(s) involved, and type of funding/donors, etc. Great care was taken to develop a sampling process that would protect the confidentiality of survey participants and their responses. A cross-sectional online survey was conducted throughout the region. The survey questions sought to collect broad observations about overall capacity needs or gaps in biorisk management systems in the BMENA study countries. It included questions about technically skilled human resources in various aspects of biosafety management systems, availability of sustainable training programs, and challenges such as lack of human resources, monetary funds, specific bio risk management skills, etc. Questions were also developed to assess capacity for biorisk management and oversight regulation, such as availability of national/institutional biosafety committees and scientist/professionals for regulatory compliance assistance. Institutional Review Board approval was obtained through the Aga Khan University in Karachi, Pakistan. Survey subjects were identified by the country coordinators.

### Subject Inclusion Criteria

The following member countries as per World Health Organization (WHO) definition of BMENA region were included in the study: Afghanistan, Algeria, Bahrain, Egypt, Iran, Iraq, Israel, Jordan, Kuwait, Lebanon, Libya, Mauritania, Morocco, Oman, Palestine, Pakistan, Qatar, Saudi, Sudan, Syria, Tunisia, Turkey, United Arab Emirates (UAE), and Yemen. Members of biosafety associations of these countries were invited by the study country coordinators to participate in the survey. In countries without biosafety associations, members of other scientifically related associations were invited. Thirty members of these various associations from each country were identified by random selection to receive the survey questionnaire *via* email.

### Analysis

Data from desktop review were entered and analyzed using Microsoft Excel (Windows XP 2008). Results were analyzed as categorical data. The biosafety initiatives reported from member countries were categorized as per the following headings: (a) general awareness sessions: activity conducted to raise general knowledge/education of biosafety and biosecurity among the scientific community; (b) human resource development: activity that translated into hands-on training of the scientists in a particular aspect of biosafety and biosecurity with an aim to transfer technology and to provide expertise at local or regional levels; (c) institutional capacity building: activity that resulted in development of network/association/foundation that would foster the biosafety and biosecurity development at the local/regional levels; (d) scientific collaborations between the member countries or with international communities which included initiatives such as regional conferences/meetings; and (e) sustainable collaborative projects at regional levels such as infectious disease surveillance, formal laboratory biosafety and biosecurity training, responsible science/bioethics and scientific cooperation, student exchange, etc. Additional information where available was recorded regarding the duration of activity, funding support, and feedback from participants of the activity.

The survey was kept open for a duration of 10 working days, and responses were directly downloaded from SM and analyzed/graphed in Microsoft Excel. The survey results were analyzed as ordinal data, responses to various survey questions were recorded as ordered categories (excellent to poor). Frequencies of the responses were generated as percentages of excellent, good, average, and poor categories of resource availability.

## Results

### Desktop Review

**Figure 1:**
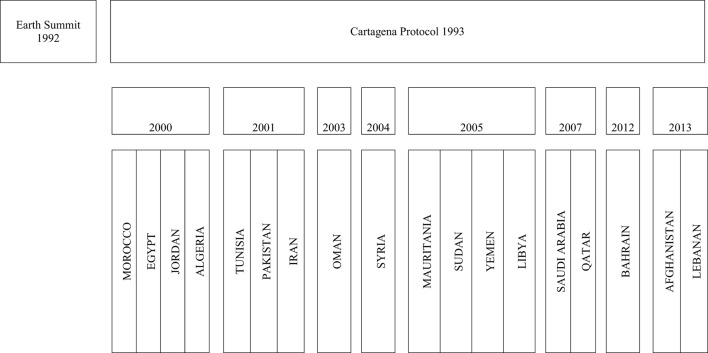
**Time lines for the biosafety initiatives in BMENA region 2001–2013**. Time lines of biosafety and biosecurity initiatives in BMENA region following Cartagena Protocol. The boxes represent year in chronological order in which biosafety initiative could be traced for the member countries by desktop review.

A total of 1832 biosafety/biosecurity initiatives were recorded from 97 web links that were reviewed, after removing the duplicate, 60 web links (1–60) were used for analysis. It was encouraging to note that every country listed in the BMENA region had some initiatives related to biosafety/biosecurity between 2001 and 2013 (Figure 1). The focus of these initiatives was found to be general awareness in the field of biotechnology and biomedical laboratories with particular interest in risk assessment and mitigation 13% (*n* = 239), followed by awareness of genetically modified organisms (GMOs) 9.17% (*n* = 168). Very little data were available for initiatives for other areas of significant biosafety concern, such as animal health sciences, chemical industry, or radiological industry.

**Figure 2:**
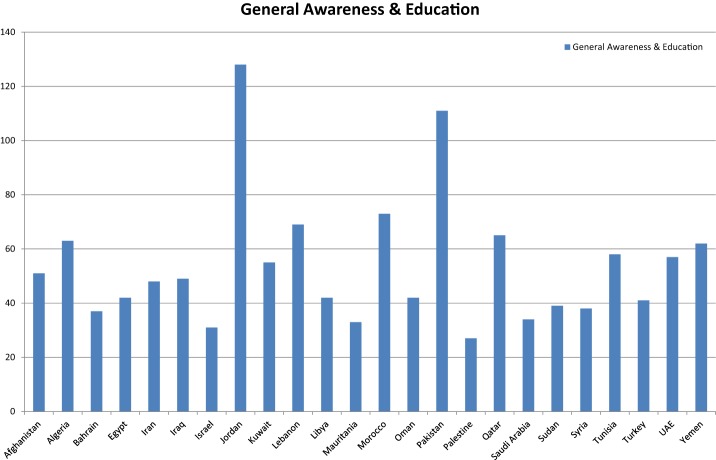
**General awareness and educational initiatives BMENA region 2001–2013**. General awareness initiatives were defined as an activity conducted to impart general knowledge of biosafety and biosecurity among the scientific community to raise general awareness. Most of these activities were conducted in form of workshops and seminars.

When categorized into types, most of the initiatives were found to be in the category of general awareness and information exchange workshops at country level 70.68% (*n* = 1295). The topics most commonly addressed in these awareness sessions included basic biosafety, biosecurity, biocontainment, and biorisk management in biomedical and biotechnology laboratories (6–39). Other aspects included bioterrorism and means to combat it, as well as bioethics and dual use resebiosafety, biosecurity, biocontainment, and biorisk management in biomedical and biotechnology laboratories (6–39). Other aspects included bioterrorism and means to combat it, as well as bioethics and dual use research of concern and other ethical dilemmas (21, 34, 38, 40–44). Scientific conferences and meetings on GMOs and other biotechnology-related topics (31, 45–49) were the next most frequently conducted initiatives at 18.5% (*n* = 339) (see Figure 2).

**Figure 3:**
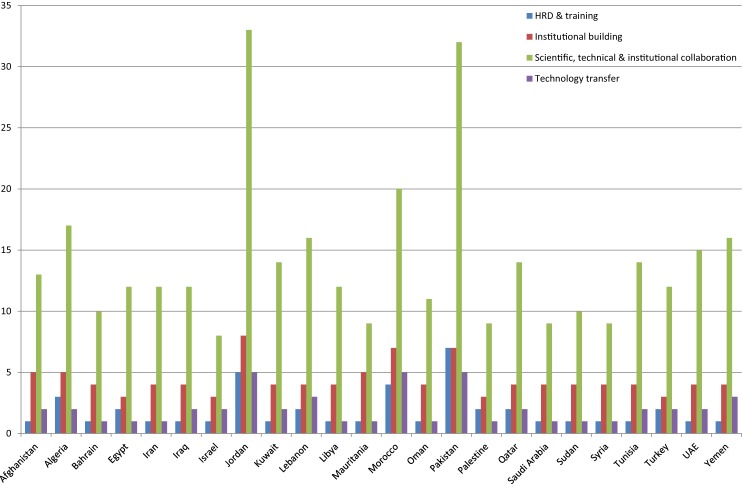
**Other biosafety initiatives in BMENA region conducted during study period 2001–2013**.

Capacity building was defined for this project as any initiative focused on technology transfer, hands-on training, and assistance in development of national framework, i.e., development of national/institutional biosafety committees or sustainable scientific projects with common regional issues and long-term measureable outcome, such as infectious disease surveillance/laboratory diagnosis, etc., in the region (Figure 3). Of the initiatives reviewed, 2.67% (*n* = 49) were in the category of hands-on training. Very few initiatives were identified using key words “student exchange program, regional training centers, and disease surveillance” against collective term BMENA, representing the region (50, 51).

An increased international donor interest was noted in the BMENA region for biosafety and biosecurity initiatives post 2000; 80% of the initiatives reviewed were funded by international donor/scientific agencies such as the United Nations WHO and the Environment Programme (UNEP), the International Council for Life Scientists (ICLS), and CRDF Global. United States sponsored/funded biosafety activities organized in 2001–2013 in the BMENA region included the Department of State Cooperative Biosafety Engagement Program (CBEP) and the American Association for the Advancement of Science-Center for Science, Technology, and Security Policy (AAAS-CSTSP).

Jordan appeared to be the hub in the region for biosafety, biosecurity, and biorisk management activities with (*n* = 179), followed by Pakistan (*n* = 162), Morocco (*n* = 109), Iraq (*n* = 68), Iran (*n* = 66), and Egypt (*n* = 60). This involved organization and hosting of national and international conferences, seminars, and training workshops on general awareness aspects of biosafety and biosecurity (Table 1).

**Table 1:** **Country list of institutes/organizations that conducted or collaborated toward biosafety and biosecurity initiatives in the BMENA region 2001–2013**.

No	Country	Organizations/institutes
1	Afghanistan	Afghan Biorisk Association
2	Jordan	El Hassan Science City (EHSC)/Royal Scientific Society (RSS)
		Middle East Scientific Institute For Security (MESIS) [earlier known as Cooperative Monitoring Centre (CMC)]
		Higher Council for Science and Technology
		Jordan University of Science and Technology/Princess Haya Biotechnology Centre
		University of Jordan/Hamdi Mango Center for Scientific Research (HMCSR)
3	Lebanon	Lebanese Agricultural Research Institute
		American University of Beirut
		Lebanese National Council for Scientific Research
4	Libya	Environment General Authority of Libya
		Libyan Association for Biotechnology
		Libyan National Committee for Bioethics, Biosafety and Biosecurity
5	Morocco	Moroccan Biosafety Association
6	Tunisia	Ministry of Higher Education and Scientific Research in Tunisia Centre of Biotechnology of Sfax Tunisia
		The Tunisian Association of Biotechnology
7	Pakistan	Pakistan Biological Safety Association
		Biological Safety Association of Pakistan
		National Task Force for Biosafety initiatives
8	Israel	Israel Biological Safety Association
9	Turkey	Biotechnology Association of Turkey
		Biosafety and Bio Economy Association of Turkey
10	Iran	Iranian Biosafety Association
11	Egypt	Egyptian Biological Safety Association

### Online Survey

The survey was administered to the life scientists identified for each participating country using the commercially available MailChimp software program within the SM platform, an internationally recognized leader in online surveying technology. SM offers state-of-the-art features in both survey design and delivery capabilities. In order to help manage panels of potential survey respondents, SM has developed a dedicated email service called MailChimp, which allows for targeted delivery of survey requests for participation.

The survey was initially released as a pilot, using Pakistan as the test group. The pilot survey was released on June 5, 2013: 50 requests to participate were sent out, with 25 participants opening the request, and 23 agreeing to participate. As per the IRB protocol, respondents were required to agree to take the survey once they understood any potential harm by participating before they could proceed to the questions within. Thus, they could open the survey, agree to the human ethics considerations, and then view the remaining questions. At this point, most respondents stopped participating. A disclaimer was introduced at this point stating that all data will be confidential and anonymous.

The final survey was released on June 30, 2013. Approximately 215 survey invitations were sent out among 11 countries (some country associations had less than 30 members). Twenty-eight recipients reviewed the survey, and 23 agreed to participate. This 11% participation rate was less than desired, although we expected a high response rate to be difficult from the countries in this study. Generally online survey responses would be expected to be in the 15–20% range, but cultural differences, possible fear of responding (despite statements declaring that all data were confidential and anonymous), and lack of experience with this type of information gathering might account for the lower rate. As a result, hard copies were posted to the country coordinators to send to their colleagues, but the response rate to the requested hard copies survey was also very poor. Iraq, Afghanistan, Sudan, Yemen, Algeria, and Syria had requested hard copies so that potential respondents would have access to all survey questions to determine if the survey was too dangerous or risky for participation. In the main release, 18% of respondents viewed the survey but did not answer the questions.

Although the response rate was lower than desired, the respondents were people with relevant background. Fifty-two percent of the respondents had worked as laboratory directors/managers in their fields and 70% of these were directly involved in biorisk management activities of their institutes. The survey results are reflective of the desktop review. Seventy-one percent of the respondents marked yes to the question about initiatives on general awareness on biosafety and biosecurity in their country. Of the respondents, 52% felt that biosafety initiatives in their countries were organized by NGOs, including professional/scientific societies, while 23.8% believed they were organized by the government ministries in their country. Fifty-two percent responded in the negative to the questions asking if the international initiatives were demand driven and sustainable.

Table 2 shows the results of direct questions related to the availability and reliability of different technical skills for biorisk management in the respective countries, on the scales of excellent to poor. The scale was defined as *excellent* where the technical expertise was easily available and considered highly reliable by the local scientists; *good* where technical expertise was available but was not very actively sought by local scientists (i.e., marginally reliable); *average* was defined as very limited or scarce local expertise and that was not very credible (i.e., marginally reliable); and unavailability of any local expertise was rated as *poor*.

**Table 2:** **Survey results of respondents from 11 member countries of BMENA region 2013–2014**.

	Excellent	Good	Average	Poor
Availability of technically skilled laboratory workers	22.5	42.5	30.0	7.5
Availability of skilled biosafety professionals or biorisk managers	10.0	22.5	27.5	40.0
Availability of scientists skilled in risk assessment for biohazards	2.5	20.0	40.0	32.5
Availability of technicians skilled in overseeing effective engineering controls (HVAC, BSC, etc.)	10.0	20.0	27.5	37.5
Availability of technically skilled professionals to oversee laboratory design	5.0	20.0	22.5	40.0
Availability of technically skilled workers for laboratory operation and maintenance	10.0	27.5	25.0	32.5
Availability of technically skilled workers for handling/transfer of GMO	5.0	17.5	22.5	50.0
Availability of technically skilled workers for handling/transfer of potentially infectious material	7.5	17.5	42.5	32.5
Availability of technically skilled animal handling workers	5.0	25.0	27.5	32.5
Availability of technically skilled workers with blood-borne pathogens	10.0	30.0	30.0	27.5
Availability of infrastructure and professional staff to implement biorisk management programs, including SOPs	10.0	22.5	22.5	37.5
Availability of accredited biorisk management training for senior scientists	5.0	7.5	20.0	55.0
Availability of accredited biorisk management training for lab directors or managers	5.0	12.5	15.0	60.0
Availability of accredited biorisk management training for university and graduate students	2.5	7.5	30.0	55.0
Availability of biorisk management training/teaching resources and materials	7.5	17.5	25.0	42.5
Availability of national/institutional biorisk management oversight, such as regulatory compliance assistance, or institutional biorisk management committees	7.5	17.5	17.5	47.5

Survey results were in accordance with the desktop review findings of biosafety initiatives during the 2001–2013 time period. We found initiatives to be largely focused on general awareness or on introductory courses on biosafety with limited human resource training and technology transfer opportunities. Consequently, there was a dearth of skilled human resources in the region as evident from survey results.

## Discussion and Recommendations

Significant advancements in the field of biotechnology in the late 1990s and early 2000s raised international concerns for biosafety related to biodiversity, particularly pertaining to risk assessment and risk mitigation regarding the impacts of new research products. These concerns were more pronounced in the agriculture field with development of Bt. cotton (GMOs) and its impacts on natural plants and the environment. These international concerns, at the forefront in the United Nations Conference on Environment and Development (UNCED), were formally recognized in what became the first Earth Summit, held in Rio de Janeiro in June 1992 (1, 2). This resulted in the Convention on Biological Diversity, from which originated the 1993 Cartagena Protocol of Biosafety. The terms biosafety and biosecurity, thereafter, gained much popularity and focus among the scientists and policy makers globally. In the BMENA region, biosafety-related initiatives can be traced back to as early as the year 2000 (1), as shown in Figure 1. We found the Cartagena protocol to be a catalyst for the serious initiatives in the capacity-building efforts in the BMENA region. However, most of the early initiatives were focused on national frameworks for biotechnology, aspects such as control GMOs and living modified organisms (LMOs), and environmental effects of agricultural products, such as Bt Cotton, perhaps because of its wider impact at the global level (2). We found 18% of total initiatives to be focused on biosafety issues related to LMOs. Although these are encouraging figures, the BMENA region is the most water-scarce and dry region worldwide. Countries across the region, especially those around the Mediterranean Sea which are highly dependent on agriculture, are tempted to use GMOs to meet consumer food demand. Therefore, more concentrated efforts are required to initiate open forums to discuss current controversies related to the pros and cons of the use of LMOs and GMOs, as well as the long-term effects on local biodiversity. The future of genetically engineered foods and crops in BMENA region will depend heavily on choices governments make regarding the regulation of this technology; therefore, coordinated and strong regional efforts are urgently required. The BMENA Organization for Economic Co-operation and Development (OECD), a forum in which BMENA governments work together to share experiences and seek solutions to common economic problems, can perhaps be an effective forum to raise the concern about the local legislations, uses, and transportation of LMOs in the region.

Post 9/11 and anthrax in the mail bioterrorism in 2001, the focus on biosafety initiatives in the BMENA region broadened to include other sciences, mainly biomedical sciences and to some extent veterinary sciences, bioengineering, chemical/nuclear sciences, and agricultural sciences, especially in countries with the backdrop of dynamic or unstable geopolitical circumstances (46–49). With the support of international organizations that focus on biosafety and biosecurity, a number of biosafety initiatives took place after 2000 in the scientific community of the BMENA region. These initiatives were primarily focused on raising awareness about biosafety and biosecurity.

Seventy percent of the total initiatives recorded fell in the category of general biosafety awareness sessions, which is an encouraging finding. However, interpreting these results at the country level is most challenging and complicated because of the diversity of the population strata, cultures, socioeconomic statuses, availability of funds, and development needs of study countries. For example, disparity is noticeable in terms of number of initiatives when compared with the per capita population of member countries. The 162 initiatives in Pakistan, the sixth most populous country in the world with an estimated population of 184.35 million, was desperately low as compared to 109 initiatives in the country of Morocco and 62 for Qatar with populations of 32 and 2.27 million, respectively. Thus, more sophisticated studies are required at individual country level for representative situational analysis.

Capacity-building activities were difficult to assess, as it is more of a conceptual approach and varies country to country depending upon needs to successfully execute the biosafety and biosecurity activities. For this, project capacity building was defined as any initiative that was focused on technology transfer, hands-on training, and assistance in development of national frameworks or sustainable scientific projects, with common regional issues and long-term measurable outcomes such as infectious disease surveillance/laboratory diagnosis. Initiatives to develop local expertise by providing hands-on training to professionals in the region were found to be significantly lacking during 2001–2013; this was also evident from the findings of the online survey. The majority of the responses to the direct questions in the survey about the availability of reliable expertise in various technical components of biorisk management were rated as average to poor. Thus, success of sustainable biosafety progress in the region demands strengthening the expert human resource training to provide the region with a wider group of local experts with sound skills in biorisk management. Such a group of skilled professionals would then be able to create guidelines and standards uniquely suited to their circumstances. Such efforts would foster a sense of ownership of guidelines; local solutions to local problems would raise the confidence and reliance on local experts by the regional scientific community.

The noticeable general trend was the international donor interest in the BMENA region in 2001–2013. Multiple donor agencies from around the globe either funded or collaborated with the biosafety initiatives in the region. However, we found that most of the efforts had been at the individual country level and not at a regional level, resulting in some duplication of efforts. For example, six separate UNEP-funded biosafety and biosecurity awareness initiatives were recorded from individual countries between January 2002 and December 2003, including Algeria, Iraq, Iran, Turkey, Egypt, and Syria. Such duplication can best be avoided in the future by working more closely with regional groups in order to coordinate efforts and focus on regional needs. Moreover, international donor funded activities related to human resource training in some study countries were coordinated through local government authorities, such as the Ministry of Agriculture of Lebanon, the Ministry of Regional Municipalities Environment and Water Resources of Oman, and the National task force in Pakistan. Successful completion of such projects was found to be dependent on political relationships between the donor and recipient country, and was often subjected to premature termination (personal communications with the country collaborators).

Another trend noticeable from the desktop review was that countries with actively functional NGOs working as Biosafety Associations, and/or those with institutes with biosafety missions, had far more initiatives on general awareness of biosafety-related issues than those without such associations. These countries hosted biosafety programs, reflecting the heightened concern in the region. Future initiatives fostering private–public partnership are strongly recommended for successful and sustainable outcomes. Successful public/private partnership models that currently exist include the International Council for the Life Sciences (a U.S.-based non-profit agency), working in collaboration with the Royal Scientific Society of Jordan (RSS), the Biosafety and Biosecurity International Consortium (BBIC, a network of concerned individuals from 22 countries in the region), and the Moroccan Biosafety Association (MOBSA) that have developed successful sustainable projects since 2005.

Conducting the online survey related to biosafety and biosecurity issues was the most complicated endeavor in this study. Obtaining statistically significant survey data in this field, and in this region, is very difficult. The participants who did respond were relevant professionals actively involved in biorisk management activities within their respective countries; thus, the responses were considered credible and are reported herein. However, the biggest challenge, and hence a limitation of this study, was the low response rate of the online survey. Regional diversity, including differences in culture, languages, dialects, and most importantly sociopolitical unrest and perception that participation in an internationally funded survey might be harmful, resulted in a sense of fear in participants despite statements declaring that all data were confidential and anonymous.

Promoting trust between funders, regional scientists, and cooperative partners, and improving open communication about intentions and objectives for the bioengagement activity (i.e., transparency) in the region is of utmost importance. Bioengagement programs can greatly benefit by incorporating such efforts into mainstream national health and science programs, such as global aid programs focused on public health: malaria, soil and water parasitism, tuberculosis, vector-borne viruses, and HIV/AIDS. These programs over the years have gained the trust of scientific individuals and national governments; these programs can be used as a bridge in strengthening regional initiatives.

## Conclusion

There has been a concerted effort to enhance the general awareness of biosafety and biosecurity in the life sciences in the BMENA region over the last decade. Our study findings suggest that to date, efforts have largely been focused on raising general awareness among the broad scientific community. Also, countries with actively functional Biosafety Associations and other scientifically related associations had far more such initiatives than those without such associations, but much duplication of efforts and inefficiencies of scale have been seen over the past decade. Continuing international donor interest providing opportunities for future assistance in the development of technical expertise can lead to development of local guidelines related to issues unique to the region. Given the differences across the region, local solutions are important. Country-level analyses of the local capacity-building needs are, therefore, recommended.

This study provides considerable vital information for those planning biorisk management initiatives in the region. Risk assessment and mitigation in life sciences research should be made known to a broader scientific audience, as much can be gained from similar expertise in other disciplines such as engineering, chemistry, and health physics. Efforts by donor nations and agencies to sustainably support these associations and provide biosafety trainings to their broad scientific communities may be the most efficient and effective way to build cooperative regional biosafety opportunities.

## Author Contributions

EK and MC conceived the idea for this project. Literature search was conducted by NA and AH, and was reviewed and analyzed by EK, JC, AE-G, KT, and MC. The survey software was developed by HG. EK analyzed survey results and overall developed the manuscript. All authors reviewed the final manuscript.

## Conflict of Interest Statement

The authors declare that the research was conducted in the absence of any commercial or financial relationships that could be construed as a potential conflict of interest.
